# Strategies for modulating the inflammatory response after decompression from abdominal compartment syndrome

**DOI:** 10.1186/1757-7241-20-25

**Published:** 2012-04-03

**Authors:** Shinil K Shah, Fernando Jimenez, Phillip A Letourneau, Peter A Walker, Stacey D Moore-Olufemi, Randolph H Stewart, Glen A Laine, Charles S Cox

**Affiliations:** 1 Department of Pediatric Surgery, University of Texas Medical School at Houston, Houston, Texas, USA; 2 Department of Surgery, University of Texas Medical School at Houston, Houston, Texas, USA; 3 Michael E. DeBakey Institute for Comparative Cardiovascular Science and Biomedical Devices, Texas A & M University, College Station, Texas, USA; 4 Department of Pediatric Surgery, University of Texas Medical School at Houston, 6431 Fannin Street, MSB 5.236, Houston, TX 77030, USA

**Keywords:** Compartment syndromes, Decompression, Laparotomy, Systemic inflammatory response syndrome, Negative pressure dressings

## Abstract

**Background:**

Management of the open abdomen is an increasingly common part of surgical practice. The purpose of this review is to examine the scientific background for the use of temporary abdominal closure (TAC) in the open abdomen as a way to modulate the local and systemic inflammatory response, with an emphasis on decompression after abdominal compartment syndrome (ACS).

**Methods:**

A review of the relevant English language literature was conducted. Priority was placed on articles published within the last 5 years.

**Results/Conclusion:**

Recent data from our group and others have begun to lay the foundation for the concept of TAC as a method to modulate the local and/or systemic inflammatory response in patients with an open abdomen resulting from ACS.

## Introduction

Management of the open abdomen is an increasingly common part of modern surgical practice. Common clinical situations that mandate the use of temporary abdominal closure (TAC) include intra-abdominal hypertension (IAH) with new organ dysfunction (abdominal compartment syndrome (ACS)), intra-abdominal sepsis without adequate source control, damage control in trauma, and mesenteric ischemia [1]. While it is difficult to estimate the prevalence or economic impact of the open abdomen, it is associated with significant issues contributing to morbidity and mortality, including development of ventral hernias, enteroatmospheric fistulas, and un-intentional protein loss [2].

The focus of this review is to detail current thoughts on the use of TAC in the management of the open abdomen, with particular attention to decompression after ACS. We review the relevant intra-abdominal related pathophysiology involved with ACS (with emphasis on the gut), the different types of TAC and evidence to support various choices. Recent data from our group and others have begun to lay the foundation for the concept of TAC as a method to modulate the local and/or systemic inflammatory response after ACS.

### Abdominal compartment syndrome

As defined by the International Conference of Experts on Intra-abdominal Hypertension and Abdominal Compartment Syndrome (World Society of the Abdominal Compartment Syndrome, http://www.wsacs.org), ACS is defined as IAH (increased intra-abdominal pressure (IAP) (> 20 mmHg)) leading to new organ dysfunction/failure [3,4]. In general, there is improvement in organ function after decompressive laparotomy. ACS can be subdivided into primary, secondary and recurrent types, depending on whether the inciting factors are abdominopelvic (primary) or in a setting free of intra-abdominal injury (secondary) [4]. Key factors in the development of primary ACS include continued hemorrhage and hemorrhagic shock from trauma, decreased space secondary to abdominal packing and bleeding, tissue edema, and translocation of fluid (third spacing) [3]. Secondary ACS is more common in settings of systemic injury (i.e., burns and or sepsis) in the setting of massive fluid resuscitation [4]. The cornerstone of management of ACS involves early decompressive laparotomy [5], but mortality from ACS remains high, especially when the diagnosis is delayed [6].

### Intra-abdominal pathophysiology involved with abdominal compartment syndrome/open abdomen: major etiological factors

Pathophysiology relevant to a discussion of TAC after ACS can be divided into several general processes including global and regional ischemia/reperfusion (IR), intestinal edema, translocation of fluid into the lumen and peritoneal cavity (third spacing), systemic neutrophil priming, and reperfusion related injury after abdominal decompression.

#### Intestinal ischemia/reperfusion

Hemorrhagic shock followed by resuscitation leads to intestinal injury by IR related mechanisms. The gut is especially susceptible to shock related reductions in blood flow secondary to both reductions in circulating blood flow as well as shock related redistribution in blood flow. Laboratory based studies have determined that the kidney, stomach, and intestines experience the greatest decrease in blood flow after hemorrhagic shock [7]. Ischemic injury in the intestine continues to persist after crystalloid based resuscitation [8].

The pathophysiology related to IR mediated gut injury is similar to that affecting the lungs and kidneys; it has been termed by some investigators as the acute intestinal distress syndrome [9]. IR results in mucosal damage and increased permeability. Mucosal damage has been attributed to numerous factors including intestinal phospholipase A2 (PLA 2) mediated arachadonic acid derived byproducts [10], mast cell infiltration and degranulation [11], epithelial cell apoptosis [12], increases in platelet activating factor (PAF) and pro-inflammatory cytokines [13], free radical mediated injury [14], and production of endothelins [15]. Consequences of these interacting factors include intestinal edema.

The increase in mucosal permeability induced by gut IR may account, in part, for distant organ injury. A large body of literature has focused on lung injury. IR mediated lung injury may represent a neutrophil mediated event. Gut derived endotoxin escape into the systemic circulation has also been implicated, potentially through a TNF-α related mechanism [16]. Other pro-inflammatory cytokines (e.g., IL-1α, IL-1β, IL-6, IL-8, IL-18, cytokine-induced neutrophil chemoattractant, and granulocyte colony stimulating factor (G-CSF)) have also been investigated as contributors, potentially by upregulating endothelial based participants in neutrophil adhesion, such as E-selectin or intracellular adhesion molecule-1 (ICAM-1) [17-20]. Membrane derived phospholipids such as PAF, have also been implicated [21], and may be involved in priming of naïve neutrophils. Investigators have also noted PLA 2 mediated arachidonic acid byproducts to participate in IR mediated lung injury [22]. Other potential mediators of distant lung injury include toll like receptors [23], oxygen derived free radicals [24], activation of alveolar macrophages [24], nitric oxide [25], activation of nuclear factor - κB [26], activation of complement [27], and production of endothelins [28]. Mesenteric lymph may represent the major conduit for gut derived mediators of distant organ injury, as will be described later in this review.

IR injury leads to inflammatory cell infiltration into the intestinal muscularis and is associated with activation of transcription factors such as nuclear factor - κB, signal transduction and activator of transcription - 3 (STAT-3), and nitric oxide, and leads to ileus i [29,30]. Ileus leads to significant consequences, including delaying enteral feeding and increasing the risk of septic complications, hospital length of stay, and health care costs [31].

#### Resuscitation strategies

Resuscitative strategies may play a role in the development of secondary ACS and ACS in the open abdomen. In particular, high volume resuscitation (in the setting of previous IR injury) may increase risk of development of ACS, potentially via exacerbation of organ edema. Multiple reports have associated high volumes of crystalloids and/or high volume of blood products to harbor increased risk for developing IAH and/or ACS [32-36]. Colloid based resuscitative strategies have been associated with decreased incidence of ACS, secondary in part to decreasing total volume of fluids administered [34]. With the increased adoption of blood product based resuscitative strategies, it is important to recognize that administration of large amounts of blood products has also been associated with an increased risk of developing ACS.

### Abdominal effects of abdominal compartment syndrome/open abdomen

#### Intestinal edema

**Figure 1:**
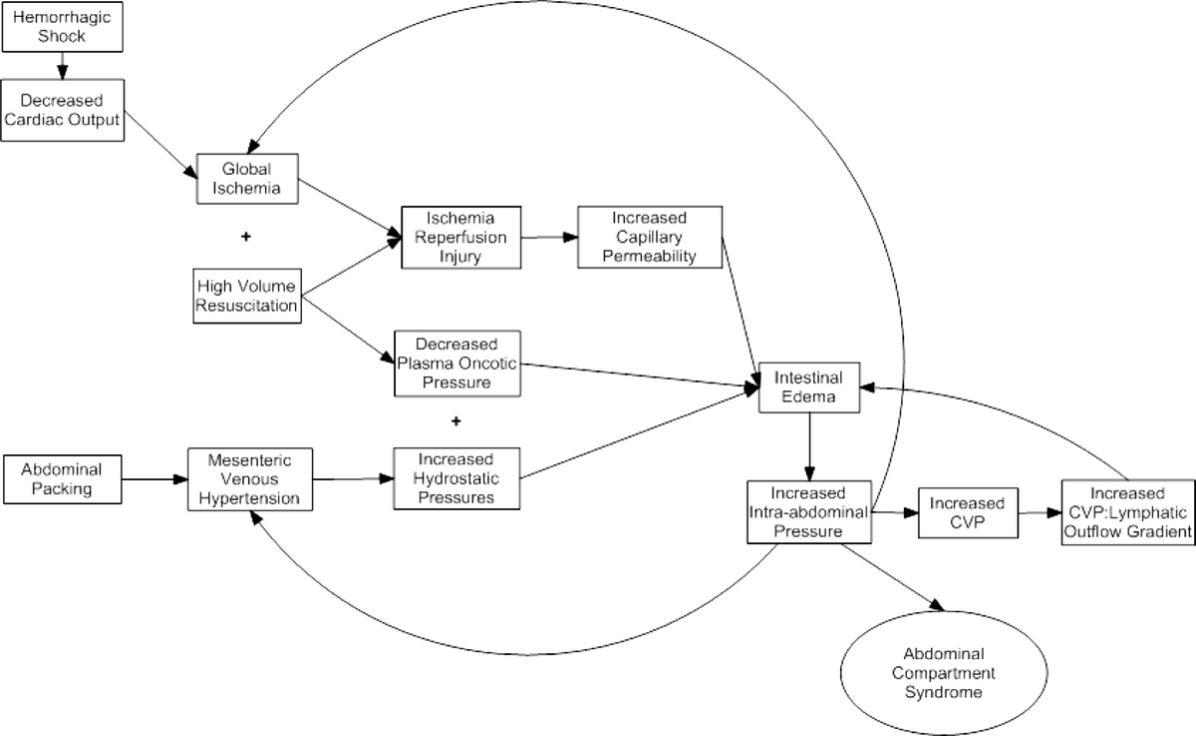
**Increased survival from traumatic injury can be attributed in part to aggressive resuscitation including early resuscitation with crystalloid and blood products and the almost universal adoption of damage control surgical procedures (i.e., abdominal packing)**. The interplay of these factors alter hydrostatic and oncotic pressure differentials contributing to the formation of hydrostatic intestinal edema. Specifically, the decrease in plasma oncotic pressures (secondary to hemodilution from resuscitation) combined with the increase in capillary hydrostatic pressures (secondary to abdominal (peri-hepatic) packing induced increases in mesenteric venous pressures) leads to net efflux of fluid into the interstitium. Contributing factors also include increased capillary permeability from IR mediated gut injury in the context of hemorrhagic shock/resuscitation and elevated central venous pressure during resuscitation which prevents lymphatic mediated efflux of fluid out of the interstitium (secondary to an elevated central venous pressure to lymphatic flow gradient. In addition, edema leading to increases in IAP can act in a feed forward manner; increases in central venous pressure (CVP) seen with increased IAPs also prevents lymphatic efflux of fluid out of the interstitium. The decrease in cardiac output, in addition to leading to further ischemic injury, can also lead to increased administration of fluids, worsening exacerbating hemodilution. Additionally, venous hypertension is a documented consequence of increasing IAPs, leading to further exacerbation of intestinal edema.

Aggressive resuscitative strategies and damage control surgical procedures alter hydrostatic and oncotic pressure differentials contributing to the formation of hydrostatic intestinal edema [33-39]. Specifically, the decrease in plasma oncotic pressures (secondary to hemodilution from resuscitation) combined with the increase in capillary hydrostatic pressures (secondary to abdominal (peri-hepatic) packing induced increases in mesenteric venous pressures) leads to net efflux of fluid into the interstitium. Contributing factors also include increased capillary permeability from IR mediated gut injury in the context of hemorrhagic shock/resuscitation [40-42], and elevated central venous pressures during resuscitation which prevents lymphatic mediated efflux of fluid out of the interstitium (secondary to an elevated central venous pressure to lymphatic flow gradient) [43,44]. The interplay of these variables is demonstrated in Figure 1.

Edema is often viewed as an inevitable consequence of resuscitation with little attention paid to its potential role as an initiator and/or propagator of dysfunctional signaling pathways. In laboratory models, intestinal edema (in the absence of neutrophil mediated injury or IR injury) serves to promote ileus, potentially by activation of signal transduction cascades (nuclear factor - κB and STAT-3), decreased phosphorylation of the regulatory myosin light chain_20_, and decreased intestinal contractility [45]. Additionally, edema alters the physical properties of the intestine, including changes in calponin, vimentin, and filamentous:globular actin, increases in interstitial pressure, and decreases in stress and residual stiffness [45]. Certain therapies that lead to improved intestinal transit (e.g., hypertonic saline) reverse physical changes induced by edema [45,46]. In addition, edema contributes to increased tissue permeability [47].

In addition to edema causing ileus, there is some evidence that ileus may lead to a cycle preventing edema resolution, secondary to interference with lymphatic mediated efflux of interstitial fluid [3,48]. Because lymphatic vessels within the bowel wall are valve-less [49], forward flow of interstitial fluid into lymphatic vessels is dependent on peristalsis. Therefore, decreased intestinal contractility may prevent efflux of excess interstitial fluid into lymphatic channels and subsequent amelioration of edema. Moore-Olufemi et al. examined the effects of primary and secondary ACS on mesenteric lymph flow and suggested that abdominal decompression may exert a beneficial effect on intestinal edema by decreasing lymphatic outflow pressures, and improving lymph mediated efflux of tissue water [50].

#### The abdomen as an inflammatory bioreactor

##### Mesenteric lymph

Gut derived lymph has increasingly been studied as a medium for systemic neutrophil priming and activation leading to distant organ injury. Early studies indicated that mesenteric lymph sampled after hemorrhagic shock activated neutrophils and was toxic to endothelial cells [51]. Given the almost immediate delivery of mesenteric derived lymph to the lungs, interest grew evaluating mesenteric lymph as the responsible agent for shock/resuscitation induced lung injury. Subsequent studies confirmed these preliminary findings and also demonstrated that mesenteric lymph represented a priming agent for naïve neutrophils, increased E and P-selectin and ICAM-1 expression on endothelial cells, and contributed to pro-inflammatory cytokine production (IL-6) [52-56].

Further demonstrating this relationship, lymphatic diversion (usually by ligation) has been shown to prevent distant organ injury [53,57]. As this does not represent a translatable therapeutic strategy in most cases, other interventions have been studied as a way to modulate post IR mesenteric lymph bioactivity, including hypertonic saline [52,58]. In addition, the apparent role of leukotriene B4 in lymph mediated lung injury may offer new avenues for pharmacologic inhibition [59]. However, novel translatable ways to affect the pro-inflammatory status of mesenteric lymph may offer promise to attenuate distant organ injury.

##### Peritoneal fluid

Factors contributing to ACS include translocation of gut derived interstitial fluid into the peritoneum. Peritoneal fluid, historically, has not been evaluated as a potential driver of systemic inflammation. Evidence from other investigators, however, indicates that peritoneal fluid may be biologically active and may represent a therapeutic target. Peritoneal fluid from burn patients contains increased levels of pro-inflammatory cytokines [60] and gut derived peritoneal fluid (accumulated during abdominal aortic aneurysm repair) increases polymorphonuclear cell expression of CD11b [61]. Increased concentrations of pro-inflammatory cytokines in peritoneal fluid has been shown to correlate with poorer outcome in burn patients with IAH/ACS [60] and in an animal model of peritonitis [62].

Peritoneal fluid and lymph are similar in that the source of both is interstitial fluid. In addition to uptake via lymphatic channels, gut derived interstitial fluid is redistributed into the gut lumen and the peritoneal cavity [63]. Adding to the potential relevance of peritoneal fluid as an initiator and/or propagator of systemic inflammation is the fact that peritoneal fluid is taken up into the systemic circulation via lymphatic (primarily) and capillary channels. This process is enhanced in the setting of inflammation [64]. Peritoneal drainage after cardiopulmonary bypass was associated with decreased serum levels of pro-inflammatory cytokines [65]. Shah et al. recently demonstrated that peritoneal fluid collected after development of ACS primes naïve neutrophils and monocytes via receptor dependent and independent pathways and that decompressive laparotomy does not significantly alter the priming capabilities of peritoneal fluid [66]. In addition, peritoneal fluid from a model of multiple organ dysfunction driven by gut IR and intra-abdominal sepsis has also been shown to prime naïve neutrophils [67]. In addition to having a potentially bioactive role, a purely mechanical role has been suggested by certain studies. Catheter drainage of ascites may prevent the need for decompressive laparotomy in certain cases of ACS [68].

##### Reperfusion syndrome after abdominal decompression

Decompressive laparotomy is widely accepted as a mandatory intervention in patients with ACS [5]. Although this procedure is associated with, often, immediate improvement in some measures of organ function, little attention is often paid to the fact that decompression may lead to release of inflammatory mediators that serve to propagate multiple organ dysfunction secondary to a reperfusion like injury [69,70]. Overt reperfusion syndrome resulting in death is rare; however, a post-decompression reperfusion syndrome is a potential reason why mortality from ACS remains high [6]. A central component of ACS associated reperfusion syndrome likely occurs secondary to repeated intestinal IR injury.

**Figure 2:**
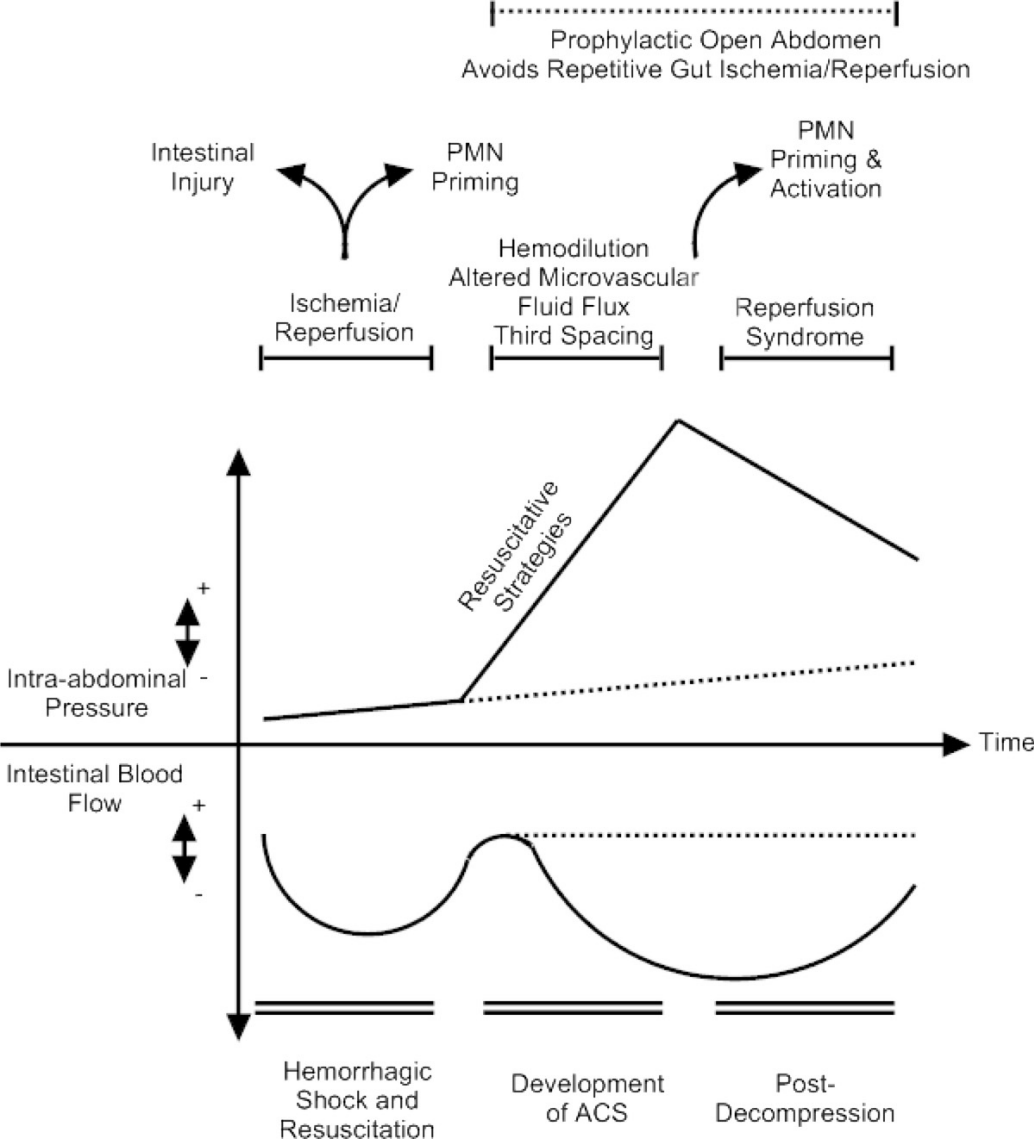
**The factors leading to abdominal compartment syndrome (ACS) are multifactorial, and include ischemia/reperfusion injury from hemorrhagic shock/resuscitation, third spacing of fluid into the gut lumen, interstitium, and peritoneum secondary to hemodilution and altered microvascular fluid flow, and polymorphonuclear (PMN) cell priming leading to distant organ injury**. Hemorrhagic shock generally has minimal effects on IAP; however, with high volume resuscitation, the interplay of increased capillary permeability secondary to ischemia/reperfusion injury and decreased oncotic pressures (secondary to hemodilution from resuscitation) can lead to a rapid increase in IAPs. While major effects are intra-abdominal, the interplay of these factors lead to distant organ injury, potentially through systemic neutrophil priming and activation. Additionally, post-decompression, a reperfusion syndrome may occur and function as a second hit. This is secondary to sudden release of flow-limiting elevated IAP, leading to recurrent gut reperfusion injury and release of pro-inflammatory mediators. Additionally, peritoneal fluid may serve as a propogator of neutrophil priming.

While most previously published animal models of ACS poorly mimic its pathogenesis [71], they are extremely useful for determining the effects of IAH, particularly in the development of intestinal ischemia. Development of ACS is associated with decreased intestinal perfusion, and these changes occur well before histological evidence of injury [72]. Decreased local perfusion persists even when global hemodynamic parameters remain within the normal range, indicating increased IAP as an inciting factor [73]. More recent work has begun to define the inflammatory response initiated by abdominal decompression. Rezende-Neto et al. demonstrated a significant increase in lung neutrophil myeloperoxidase, IL-6, and TNF-α after decompression from a period of ACS, suggesting decompression as an inciting event for injury [74]. This has been replicated in other investigations; Oda et al. demonstrated a similar exacerbation of cytokine levels and lung injury when evaluating a period of induced ACS after hemorrhagic shock/resuscitation induced injury, with levels and injury continuing after cessation of the period of ACS (i.e., decompression) [75]. Prior hemorrhagic shock/resuscitation induced injury results in a lower tolerance for increasing IAPs with regard to end organ injury [76]. Although ACS is not classically viewed as an initiator of intestinal IR injury, the data presented do serve to support this notion. Indeed, artificially induced ACS and subsequent decompression has been used as a model for the study of kidney IR injury and recently, to mimic intestinal IR injury [77,78]. Additionally, the published data on decompression leading to increased systemic neutrophil activation and continued bioactivity of peritoneal fluid, as described earlier, serve to support the notion that decompression after ACS may serve as a continued driver of multiple organ dysfunction. This data also suggests that prophylactic use of the open abdomen in certain high risk situations may affect the course of injury by preventing a second IR injury "hit." The interplay of IR, resuscitation, development of ACS, and reperfusion syndrome after decompression is demonstrated in Figure 2.

### Temporary abdominal coverage

Having elucidated major pathophysiological processes that contribute to the development of ACS and dysfunction post-decompression, we now turn our focus to TAC. We will briefly review the types of TAC and the current clinical evidence supporting the use of various forms of TAC. We focus on studies evaluating ACS and related processes and evidence that support the notion that TAC may modulate systemic inflammatory processes.

#### Choices for temporary abdominal closure

Choice for TAC can broadly be divided into non negative pressure and negative pressure therapies.

##### Non negative pressure therapies

The technique of planned re-operation for intra-abdominal pathology has been described since the 1980's. The technique was first widely adopted in the management of severe intra-abdominal trauma [79]. One of the earliest methods of temporary closure was accomplished by using towel clips to close the skin. Although this was fast, cheap and minimized thermal and fluid losses, there were significant disadvantages, including the inability to visualize the wound, possible evisceration between clips, damage to skin, and a higher incidence of ACS due to decreased peritoneal reserve volume [80]. Increasing peritoneal cavity volume via the use of an artificial mesh is an option for TAC. There are several types of mesh that may be used, including polyglycolic acid (Vicryl™), polypropylene (Marlex™), or polytetrafluoroethylene (PTFE), and mesh closure techniques have been combined with zipper closure techniques to allow for serial examination of bowel [81-83]. Other options including other absorbable meshes (e.g., polyglycolic acid (Dexon)), biologic products, velcro-type closures (i.e., Wittmann Patch), and silastic sheeting have been described in the literature [84-88]. Mesh closure techniques have decreased in popularity secondary to adherence of bowel to mesh and potential for injury when taking down for re-exploration and potential for fascial trauma and necrosis (especially with repeated exploration and re-suturing) [81].

Another method of TAC is the Bogota bag, named by Mattox while observing surgery for trauma in Colombia. This method utilizes an intravenous fluid bag sewn to the skin to create a translucent silo to expand the effective volume of the abdominal cavity [89]. Advantages of this technique include speed, cost, and the ability to serially inspect abdominal contents. Initial series of TAC used the Bogota bag widely [90]; however, more recent literature indicates that this technique may associated with increased loss of fascial domain [80]. Modifications including sewing it to the fascia and progressive tightening of the bag with decreasing intra-abdominal edema have been described in an attempt to prevent retraction and increase likelihood for eventual fascial closure [91].

These techniques are associated with bowel fistula formation, retraction of the abdominal fascia, and intestinal adherence to the prosthesis. Lack of hermetic closure and efficient drainage can frequently cause profuse leakage of ascites, unpleasant nursing care and issues with fluid management.

##### Negative pressure therapies

The first reported negative pressure assisted form of TAC was described by Schein et al and described for the use of abdominal sepsis. The "sandwich" technique utilized a piece of mesh sewn to the fascia with surgical drains overlaying and coverage with an adhesive dressing [92]. The vacuum pack method was a derivation of this technique. Utilizing commonly available materials (a perforated polyethylene sheet to protect peritoneal contents, surgical towels, silicone drains, and an adhesive plastic drape), an inexpensive negative pressure assisted method can be quickly fashioned. This technique was initially developed to prevent fascial trauma and to facilitate nursing care [81,93,94]. Various modifications of these techniques have been described [83,95-97].

Another popular negative pressure TAC device is the Vacuum Assisted Closure (VAC, Kinetic Concepts, Inc, San Antonio, TX) therapy system. The system requires the placement of a non-adherent perforated plastic barrier over the intra-abdominal viscera, followed by coverage with a polyurethane sponge and sealing with a plastic sheet. An aspiration system is connected to the sponge and a suction and negative pressure applied [98]. Many modifications of the VAC closure technique have been described [99,100]. In addition, new commercially available negative pressure dressings have recently been introduced (ABThera, Kinetic Concepts, Inc, San Antonio, TX; RENASYS Open Abdomen Solution, Smith and Nephew, St. Petersburg, FL)

#### Evidence for temporary abdominal closure techniques

Although there are a number of individual series describing individual institution experiences with varying forms of TAC, the focus of this review will be on series comparing differing forms of TAC to move towards an evidence based paradigm for choice of TACs in differing clinical situations. There are no prospective, randomized trials demonstrating superiority of any particular TAC technique.

##### Fascial closure

There are few randomized trials comparing differing forms of TAC and eventual outcome with regards to fascial closure. Bee et al. conducted a randomized trial comparing VAC therapy to polyglactin mesh and demonstrated that no significant difference in delayed primary fascial closure rates, fistula formation (21% versus 5%, VAC versus polyglactin mesh, respectively), or intra-abdominal abscess formation. It is important to note that the overall rate of fascial closure (approximately 30%) is low when compared to other series, and may have been secondary to the patient population at the institution [101]. A recent meta-analysis evaluating negative pressure therapy (VAC, vacuum pack), Whitman patch, mesh closures (including mesh, zipper, and silo), skin closure, and retention sutures demonstrated that the Whitman patch, VAC therapy, and use of retention sutures led to the highest rates of delayed fascial closure [89].

##### Abdominal compartment syndrome in the open abdomen

As indicated earlier, the fluidity of indications for the open abdomen, especially in the setting of abdominal trauma and risk factors known to harbor an increased risk of IAH, has led to significant pre-emptive use of TAC in an attempt to prevent development of ACS. Despite this, there is an entity that deserves attention; namely development of ACS in the open abdomen. Although there is scant literature on this topic, currently published evidence suggests that vacuum assisted TAC may harbor an increased risk of recurrent IAH and development of recurrent ACS. Gracias et al. published a series of development of ACS in the open abdomen and reported 5 cases - in all cases, ACS developed within 12 hours post-operatively and the vacuum pack dressing was utilized as TAC [32]. Two recent laboratory papers also suggest that negative pressure therapy decreases abdominal wall compliance and may harbor a theoretical increased risk of IAH [102,103]. It is important to note that in the Gracias series, 15 other patients were treated with the vacuum pack dressing without development of ACS and that there are currently no published prospective studies evaluating the effect of early application of vacuum assisted therapies in the setting of decompression from ACS.

##### Mortality and outcome - clinical studies

A recent meta-analysis of series of differing TAC techniques demonstrated that VAC therapy and the Whitman patch were associated with the lowest mortality rates [89]. Series comparing outcome between TAC are almost universally retrospective. VAC therapy has been associated with improved early control of intra-abdominal pressure and normalization of lactate, faster time to fascial closure and ventilator weaning, and decreased intensive care unit and hospital length of stay when compared to Bogota bag closure for decompression from ACS. It is important to note in this series, although the study of patients treated with VAC was prospective, the control group was a retrospectively evaluated cohort at the beginning timepoint of the study. Although this study provides evidence that VAC therapy may have a global protective effect, other advancements in therapy and resuscitation practices over time may have accounted for the improved outcomes [104].

##### Laboratory data

Although the majority of surveyed trauma surgeons used negative pressure assisted TAC [105], there are no prospective randomized trials demonstrating superiority in the setting of decompression from ACS. A careful examination of current laboratory data is therefore imperative. We reviewed earlier that recurrent ACS in the open abdomen was associated in one series with use of the vacuum pack technique. Additionally, Benninger et al. demonstrated that negative pressure therapy was associated with the lowest abdominal volume reserve capacity (i.e., lower volume of fluid resulted in a greater increase in IAP) in in-vitro and in-vivo models. The authors theorized that, in the setting of decompression from ACS, these therapies may place one at a higher risk for recurrent IAH [102,103]. These data mandate further study into the safety of negative pressure therapy in the immediate post-decompression period.

There are few pre-clinical studies evaluating negative pressure therapy in relevant large animal models. Kubiak et al. recently compared VAC therapy with negative pressure turned on to VAC therapy with negative pressure off in a large animal model of multiple organ dysfunction driven by gut IR and intra-abdominal sepsis. Notable findings included decreased mortality in the negative pressure group along with significant amelioration of lung injury. In addition, decreased systemic concentrations of pro-inflammatory cytokines were noted [106] In a clinically severe large animal model of non-infectious ACS, Shah et al. recently demonstrated that immediate post-decompression application of negative pressure therapy as compared to Bogota bag closure was associated with no increase in recurrent IAH or worsened outcomes with regards to physiological variables, organ edema, and organ histology. Given the small sample sizes, no conclusions could be made regarding survival. Negative pressure therapy was associated with reductions in central venous and mesenteric venous pressures which over time, may be associated with more favorable fluid flux profiles and augmented resolution of intestinal edema [66,107,108]. These data demonstrate that negative pressure therapy appears safe in the immediate post-decompression period from ACS and may be associated with improved outcomes/organ injury parameters.

## Conclusions/future study

The literature on ACS and management of the open abdomen clearly demonstrate that prevention is the best cure. The consequences of managing an open abdomen are extensive; the entirely new surgical subspecialty of complex abdominal wall reconstruction has emerged to deal with some of these issues. Recent studies have indicated that damage control laparotomy and the use of TAC is, in some cases, overutilized [109]. Increased recognition of the causative factors and modifications in resuscitation strategies are serving to decrease the incidence of ACS, need for decompressive laparotomy, and consequent need for the open abdomen and TAC. We emphasize the point that prevention of the open abdomen represents a far superior strategy for patient management than any form of TAC for an open abdomen

There is a lack of randomized clinical trials evaluating the effects of a variety of TAC choices in the post decompression period from ACS. Recent laboratory data, however, suggest that negative pressure therapy may be associated with a global protective effect. Potential mechanisms of benefit are currently not well understood, however recent data on the pro-inflammatory nature of peritoneal fluid in infectious and non-infectious states and the role of mesenteric derived lymph in the systemic inflammatory process offer a basis for future study.

It is well documented that peritoneal fluid can be reabsorbed into the systemic circulation, primarily via lymphatic conduits [64,110]. Given this data and evidence that peritoneal fluid is pro-inflammatory by representing a potential primer for naïve neutrophils, a potential mechanism for systemic effects of peritoneal fluid can be proposed, either as an initiator (i.e., intra-abdominal sepsis) or propagator of systemic inflammation. Abdominal cavity lymph has been widely studied as a mediator of distant lung injury. Laboratory studies have demonstrated that thoracic duct lymphatic diversion ameliorates lung injury in the setting of gut IR [111]. Part of the composition of thoracic duct derived lymph is reabsorbed peritoneal fluid. Given data demonstrating that negative pressure therapy ameliorates lung injury, peritoneal fluid may increase the pro-inflammatory characteristics of lymph given its major route of uptake being diaphragmatic and peritoneal cavity based lymphatic channels [106]. One potential explanation for these observations is that augmented removal of peritoneal fluid decreases the pro-inflammatory characteristics of abdominal-activity derived lymph resulting in decreased lung injury. Further study is necessary to confirm the hypothesis that negative pressure therapy may be a method to decrease the pro-inflammatory nature of abdominal cavity derived lymph.

While the preponderance of data does not provide definitive guidelines for the management of the open abdomen post-decompression from ACS, there is a growing volume of literature that suggests that the intra-abdominal cavity may be a way to modulate outcome. Preliminary data suggests that negative pressure therapy may be associated with a global protective effect and this may be based via an effect on peritoneal fluid. Further elucidation of this mechanism may allow for improved patient outcomes by appropriate choice of TAC.

## Abbreviations

TAC: emporary abdominal closure; IAH: intra-abdominal hypertension; IAP: intra-abdominal pressure; ACS: abdominal compartment syndrome; IR: ischemia/reperfusion; PLA 2: phospholipase A2; PAF: platelet activating factor; IL: interleukin; TNF: tumor necrosis factor; G-CSF: granulocyte colony stimulating factor; ICAM: intracellular adhesion molecule; STAT: signal transduction and activation of transcription; PTFE: polytetrafluoroethylene; VAC: vacuum assisted closure.

## Competing interests

Dr. Cox has received past research funding from Kinetic Concepts, Inc.

## Authors' contributions

All authors participated in the conception, design, acquisition, analysis and interpretation of data, and drafting and critically revising the manuscript. All of the authors have approved the final submission of this manuscript.
